# Stem Cells from Dental Sources: Translational Applications in Medicine and Novel Approaches

**DOI:** 10.3390/ijms23084308

**Published:** 2022-04-13

**Authors:** Marco Tatullo, Maria Giovanna Gandolfi

**Affiliations:** 1 Department of Basic Medical Sciences, Neurosciences and Sense Organs, University of Bari Aldo Moro, 70124 Bari, Italy; 2 Honorary Senior Clinical Lecturer, University of Dundee, Dundee DD1 4HR, UK; 3 Founding Member of MIRROR—Medical Institute for Regeneration and Repairing and Organ Replacement, Interdepartmental Center, University of Bari Aldo Moro, 70124 Bari, Italy; 4 Laboratory of Biomaterials and Oral Pathology, Department of Biomedical and Neuromotor Sciences, School of Dentistry, University of Bologna, 40125 Bologna, Italy; mgiovanna.gandolfi@unibo.it

Recently, regenerative medicine has been attracting interest from scientific groups working on translational applications of applied medical sciences. In ideal conditions, the main players of tissue regeneration are stem cells, scaffolds, and intercellular signals, such as growth factors. The search for new biological sites that are easily accessible and rich in stem cells has led to the discovery of numerous novel types of stem cells, alternatives to those considered to be the “gold standard” [1,2]. Recently, oral tissues have been investigated as a smart source of stem cells; in fact, current in vitro and in vivo publications show numerous benefits of using dental stem cells for hard and soft tissue regeneration [3]. In addition, growing evidence has demonstrated that stem cells derived from subjects affected by genetic disorders can be used as in vitro “disease models” capable of investigating the cellular and molecular aspects of several disease-linked genetic alterations [4].

Mesenchymal stem cells (MSCs) from dental sources have been obtained through the analysis of several oral tissues, such as MSCs isolated from human periapical cysts, and termed “human Periapical Cyst-MSCs” (hPCy-MSCs). Nevertheless, increasing research is focussing on the alternative use of extracellular vesicles [5] to induce regeneration among cells and between different tissues interacting at the interface [6,7]. Teeth are often subjected to severe pathologies affecting dental pulp: the regeneration of vascularized pulp-like tissue has been achieved using MSCs in animal and human models, creating numerous expectations on dental-derived MSCs for dental and pulp regeneration [8]. 

Human dental pulp stem cells (DPSCs) are MSCs isolated from dental pulp. They have high proliferative potential and are easy to manage and store. DPSCs have been investigated for the regeneration of several tissue types, including bone [9]. Moreover, DPSCs’ storage has been demonstrated to be easy and safe; in fact, DPSCs do not compromise on their stemness, viability, proliferation, or differentiating capabilities, even after one year of uncontrolled cryopreservation at −80 °C [10].

Nonetheless, the oral environment is rich in MSC sources that are directly involved in several biological and functional activities related to their tissue of origin. The periodontal ligament (PDL) hosts the human periodontal ligament-derived mesenchymal stromal cells (hPDL-MSCs), which are fully involved in a number of biological mechanisms, such as the production of matrix metalloproteinases (MMPs). The crosstalk among hPDL-MSCs and specific local bioactive factors seems to be responsible for different MSC behaviors, leading to different biological processes. Interestingly, Interleukin (IL)-1β, one of the most abundant inflammatory mediators, has been demonstrated to affect the expression of MMPs and their local inhibitors, guiding the clinical appliance of orthodontic forces [11]. Moreover, recombinant human BMP-2 (rhBMP-2), one of the most recognized osteogenic factors, has been demonstrated to control the regeneration of cementum and PDL by using engineered hPDL-MSC sheets, showing how such cells could be an effective strategy for the regeneration of two different tissues, both residing within the periodontal complex [12]. 

Stem cells from dental sources (DSCs) are consistently involved in translational applications, not only in dentistry, but also in medicine. The most promising applications of dental stem cells in medicine are related to several clinical conditions. As an example, preclinical research has demonstrated that DPSC transplantation is expected to promote the recovery of ischemic stroke, by improving neurological functions and reducing the infarct size [13]. Dental stem cells have been found to be attractive for cell-based strategies for Duchenne muscular dystrophy (DMD), an inherited syndrome affecting the bones and muscles, because of their immunosuppressive properties [14]. Dental pulp also contains an MSC population that differentiates into hepatocytes, supporting and enhancing the in vivo organ regeneration [15]. DSCs have thus been used in several clinical protocols to induce or improve tissue healing thanks to their regenerative and immunomodulatory properties. In recent years, the same therapeutic effects shown by DSCs have been obtained with their exosomes [6,7]: compared to DSCs, DSC-related exosomes have interesting drug loading capacity, nanoscale size, high site-specificity, and low local-immunogenicity, together with a minimal number of side effects; such peculiarities have been used for in vivo application on several cancer animal models, showing therapeutic capacities that have important translational benefits [16]. 

It is strategic not to underestimate the important achievements obtained with these MSCs thanks to the novel and smart approaches, as such protocols may be the next way to regenerate tissues in the future. This Special Issue has robustly explained how different research fields have been opened by several researchers working with dental stem cells. A multidisciplinary approach, an increase in knowledge related to stem cell manipulation, and a futuristic rethinking of the academic approach to dental sciences, perhaps with a greater presence of biomedical and translational courses [2], could be the perfect overlapping that will take maximum advantage of increasing innovation in healthcare.
